# A Novel, Orally Delivered Antibody Therapy and Its Potential to Prevent *Clostridioides difficile* Infection in Pre-clinical Models

**DOI:** 10.3389/fmicb.2020.578903

**Published:** 2020-09-22

**Authors:** April K. Roberts, Hannah C. Harris, Michael Smith, Joanna Giles, Oktawia Polak, Anthony M. Buckley, Emma Clark, Duncan Ewin, Ines B. Moura, William Spitall, Clifford C. Shone, Mark Wilcox, Caroline Chilton, Rossen Donev

**Affiliations:** ^1^Toxins Group, National Infection Service, Public Health England, Porton Down, United Kingdom; ^2^Leeds Institute of Medical Research, Faculty of Medicine and Health, University of Leeds, Leeds, United Kingdom; ^3^MicroPharm Ltd., Newcastle Emlyn, United Kingdom; ^4^Department of Microbiology, Leeds Teaching Hospitals NHS Trust, Leeds General Infirmary, Leeds, United Kingdom

**Keywords:** *Clostridioides difficile* infection (CDI), immunotherapy of CDI, oral antibodies, formulation protecting antibodies from digestion/inactivation, *in vivo* hamster model of CDI, *in vitro* human gut model of CDI

## Abstract

*Clostridioides difficile* infection (CDI) is a toxin-mediated infection in the gut and a major burden on healthcare facilities worldwide. We rationalized that it would be beneficial to design an antibody therapy that is delivered to, and is active at the site of toxin production, rather than neutralizing the circulating and luminal toxins after significant damage of the layers of the intestines has occurred. Here we describe a highly potent therapeutic, OraCAb, with high antibody titers and a formulation that protects the antibodies from digestion/inactivation in the gastrointestinal tract. The potential of OraCAb to prevent CDI in an *in vivo* hamster model and an *in vitro* human colon model was assessed. In the hamster model we optimized the ratio of the antibodies against each of the toxins produced by *C. difficile* (Toxins A and B). The concentration of immunoglobulins that is effective in a hamster model of CDI was determined. A highly significant difference in animal survival for those given an optimized OraCAb formulation versus an untreated control group was observed. This is the first study testing the effect of oral antibodies for treatment of CDI in an *in vitro* gut model seeded with a human fecal inoculum. Treatment with OraCAb successfully neutralized toxin production and did not interfere with the colonic microbiota in this model. Also, treatment with a combination of vancomycin and OraCAb prevented simulated CDI recurrence, unlike vancomycin therapy alone. These data demonstrate the efficacy of OraCAb formulation for the treatment of CDI in pre-clinical models.

## Introduction

*Clostridioides difficile* infection (CDI) is a leading cause of infectious antibiotic associated diarrhea and a major burden on healthcare facilities worldwide (Wiegand et al., 2012; Lessa et al., 2015; Guh et al., 2020). Disruption to the normal intestinal microbiota (e.g., by antibiotic exposure) is a key risk factor for CDI, leading to loss of colonization resistance and creating a niche which *C. difficile* can exploit. CDI is a toxin mediated disease, with the primary disease–causing factors identified as two large protein toxins, Toxin A (TcdA) and Toxin B (TcdB) (Lyerly et al., 1985). A third toxin which is likely to contribute to the disease (Gerding et al., 2014), binary toxin (CDT), has been identified in a number of epidemic strains, however, CDT itself does not appear to be sufficient to cause CDI in animal models (Kuehne et al., 2014).

Currently, the standard treatment for CDI is antibiotic therapy (use of metronidazole, vancomycin and more recently, fidaxomicin), which has limited long-term efficacy, with approximately 20–30% of patients experiencing recurrent disease (Kelly, 2012) and fidaxomicin therapy resulting in ∼12% less recurrences compared to vancomycin treatment (Mullane, 2014). Alternative therapies directed at neutralizing TcdA and TcdB and restoring the disrupted microbiome are in development. These include antibody-based therapies, vaccines and fecal microbiome transfer (FMT) and are summarized by Madoff et al. (2019). Bezlotoxumab developed by Merck is the first monoclonal antibody approved to prevent the recurrence of *C. difficile* infection (Lee et al., 2017). It should be noted that despite the promising benefits reported for FMT, the transmission of an extended-spectrum beta-lactamase producing *Escherichia coli* to two patients in two independent clinical trials has been reported. Both patients developed bacteraemia and one died. Therefore, the screening of FMT donors requires improvement (DeFilipp et al., 2019).

It is likely that the host immune response is a critical factor in recurrent disease, with naturally occurring toxin antibodies shown to be protective against recurrent CDI (Kyne et al., 2001). Human monoclonal antibodies (MAbs) against TcdA and TcdB (actoxumab and bezlotoxumab) have been developed (Babcock et al., 2006) and systemic administration of these antibodies alongside standard of care therapy has been tested in two large international multicentre, double blind randomized placebo controlled trials (Wilcox et al., 2017). Administration of bezlotoxumab led to significantly lower rates (∼11% less as an absolute value compared to placebo) of recurrent disease 12 weeks after administration. Administration of actoxumab alone showed no clinical benefit, and there was no additional advantage of a combination of actoxumab and bezlotoxumab compared with bezlotoxumab alone (Wilcox et al., 2017). Thus, the therapeutic use of some MAbs against *C. difficile* toxins has been shown to be beneficial in reducing cases of recurrent disease when administered systemically. This MAb-based therapy has been licensed for the prevention of recurrent CDI (when used with standard of care antibiotics) and marketed as Zinplava.

Previously, we have described the use of systemically delivered ovine polyclonal antibodies raised against toxoids of TcdA and TcdB and against non-toxic recombinant fragments of TcdA (TxA4) and TcdB (TxB4) as a potential treatment for severe CDI. Importantly, these polyclonal antibodies can neutralize toxins from several circulating *C. difficile* toxinotypes (0, III, V) (Roberts et al., 2012; Maynard-Smith et al., 2014). In the present study, we describe the potential of these antibodies for the management of CDI when delivered as an oral therapy. The antibodies could be used alongside standard of care antibiotics and in preference to or in combination with FMT for the treatment of recurrence. This oral therapy also has the potential to be used as a prophylactic in high risk patients when entering a healthcare facility.

For toxin-mediated infections in the gut such as CDI, it would be preferable to design an antibody therapy that is delivered to and is active at the site of toxin production. The rationale for delivering the antibodies orally is to target directly the site of infection where TcdA and TcdB are released, rather than neutralizing the toxins in the circulation and gut lamina propria after significant damage of layers of the intestines has occurred. Furthermore, especially when mucosal inflammation is absent, only a negligible proportion of immunoglobulins injected systemically may reach the lumen of the gut by transudation from serum (Shearman et al., 1972). The development of several orally delivered antibody-based therapies for CDI have been described previously. These therapies include toxin-neutralizing antibodies, for example, chicken IgY (Kink and Williams, 1998) and IgG in bovine colostrum (Sponseller et al., 2015; Hutton et al., 2017).

Using platform technologies established by MicroPharm and Public Health England (PHE), we have developed OraCAb, a novel orally delivered ovine polyclonal antibody product targeted toward *C. difficile* toxins. OraCAb has several advantages over a therapy based on bovine colostrum. For example, there is a constant supply of hyperimmune serum from immunized sheep from which to prepare the product. In addition, OraCAb contains highly efficacious toxin-neutralizing antibodies purified from serum. Furthermore, bovine colostrum derived antibodies, currently used for trials, are not protected against denaturation and digestion by acidic gastric juice and pepsin in the stomach, and trypsin and chymotrypsin in the small intestine. OraCAb has been formulated to maintain the integrity of the antibodies whilst the therapeutic is passing through the stomach and small intestine. Unlike in intravenous administration, polyclonal antibodies are not expected to cause a significant systemic humoral immune response when taken orally. A recently published study (Crowe et al., 2018) demonstrated that a small amount of orally administered antibodies gain access to the lamina propria and serum only when intestinal lesions are in place, with antibody concentrations increasing with disease severity. OraCAb is intended for treatment of mild to moderate CDI where severe lesions are unlikely to occur. Neutralization of TcdA and TcdB at the site of their production will eliminate further damage in the colon and should minimize infiltration of orally administered polyclonal immunoglobulins. The major advantages of using polyclonal rather than MAbs against TcdA and TcdB are the greater avidity of polyclonal antibodies, more efficient neutralization of the very large toxin molecules and their ability to neutralize a variety of toxinotypes of TcdA and TcdB.

This work describes the development strategy used to formulate OraCAb and its potential to prevent CDI and CDI recurrence in pre-clinical *in vitro* and *in vivo* models.

## Materials and Methods

### Study Design

This study was designed to examine the protective efficacy of an ovine antibody-based, orally delivered therapeutic for the treatment of CDI, both *in vitro* and in the Golden Syrian hamster model. The antibody formulation, OraCAb, was developed to protect the antibodies during transit through the gut and to be suitable for oral administration to hamsters. The formulations were thoroughly tested *in vitro* before testing *in vivo*. The *in vivo* model was employed to demonstrate the efficacy of a variety of OraCAb formulations. The animals used in these studies were Golden Syrian hamsters obtained from a UK Home Office approved breeder (Envigo). The animals were 7–9 weeks old at the start of an experiment, weighing 80–100 g.

The Project Licenses (30-3042 and P66F2D25B) enabling these studies were approved by the Ethical Review Process of Public Health England (PHE), Porton, Salisbury, United Kingdom and the Home Office, United Kingdom. Animals were housed as single sex pairs according to the Home Office Code of Practice for the Housing and Care of Animals Bred, Supplied or Used for Scientific Purposes, December 2014 and the NC3Rs (National Centre for the Replacement, Refinement and Reduction of Animals in Research).

The collection and use of stool samples from healthy adult individuals in the *in vitro* human gut model was approved by the University of Leeds School of Medicine Research Ethics Committee (Ref: MREC15-070).

### Production of Ovine Anti-TxA4 and Anti-TxB4 Antisera and Antibodies

The recombinant antigens, TxA4 and TxB4 with sequences obtained from VPI10463 strain, were expressed and purified as described by Maynard-Smith et al. (2014). For antiserum production, sheep were immunized at 0, 4, 8, 12, and 16 weeks with 250 μg of either antigen per dose as described in Roberts et al. (2012). Pre-bleeds were collected at week 0 and test bleeds collected at 6, 10, and 14 weeks. A larger bleed was collected at 18 weeks when antibody toxin neutralization titers were high. Prior to immunization, the antigens were treated with formaldehyde, as previously described (Maynard-Smith et al., 2014).

### Purification of Bowman–Birk Protease Inhibitor (BBI) From Lima Beans

BBI was produced at PHE from commercially available Lima beans. Briefly, Lima beans were blended to a flour and defatted with ethanol. BBI was purified through a combination of aqueous acid extraction, ammonium sulfate precipitation, differential solubilization in ethanol solutions, immobilized metal affinity and anion exchange chromatography. Approximately 2.6 mg of inhibitor, >95% pure, was obtained per gram of Lima beans. The inhibitor was dialyzed against pH 7.5 HEPES (10 mM), NaCl (100 mM), sterile filtered and freeze dried for inclusion in OraCAb formulations, as required. Additional information on BBI purification is available in Supplementary Materials.

### Preparation of OraCAb Formulations

Series of OraCAb formulations used to optimize the anti-TxA4: anti-TxB4 IgG ratio, IgG concentration and comparison of protease inhibitors are given in Table 1.

**TABLE 1:** OraCAb formulations used in *in vivo* studies.

**Study**	**OraCAb for *in vivo* studies (concentration mg/ml)**		
	
	**Anti-TxB4 IgG**	**Anti-TxA4 IgG**	**Protease Inhibitor (dried egg white [DEW] or Bowman– Birk inhibitor [BBI])**
A *(in vivo)*			
DEW (1:1 B:A)	75	75	DEW 60
DEW (3:1 B:A)	112.5	37.5	DEW 60
BBI (3:1 B:A)	112.5	37.5	BBI 10
B/D *(in vivo)*			
No DEW 150 mg/ml	112.5	37.5	DEW 0
150 mg/ml	112.5	37.5	DEW 60
100 mg/ml	75	25	DEW 60
50 mg/ml	37.5	12.5	DEW 60
C *(in vivo)*			
B only	112.5	0	DEW 60
3:1 B:A	112.5	37.5	DEW 60
1:1 B:A	75	75	DEW 60
Additional ingredients			
Magnesium hydroxide (23.4 mg/ml), Dried aluminum hydroxide (26.4 mg/ml), Methylparaben (E218) (2 mg/ml), Propylparaben (E216) (0.6 mg/ml), Glycine (15 mg/ml), Simethicone (16.9 mg/ml), Sodium saccharin (0.4 mg/ml), and Mannitol (21 mg/ml).			

Anti-TxA4 IgG and anti-TxB4 IgG were formulated in 20 mM sodium citrate buffer (pH 6.0) containing 153 mM NaCl (SCS). After mixing the two antibodies in the required ratios (as described in Table 1), all other constituents of the OraCAb formulation were added and a stable suspension was prepared by mixing with a ONE-ST-C laboratory scale propeller mixer (Joshua Greaves & Sons Ltd, United Kingdom). The final volume was adjusted with SCS. For the *in vitro* gut model studies, antibodies formulated in SCS buffer only were used.

### *N*α-Benzoyl-DL-Arginine-*p*-Nitroanilide (BAPNA) Assay for Activity of Trypsin Inhibitors

The activity of the BBI and dried egg white (DEW) trypsin inhibitors was determined by analysis of the reaction with BAPNA as a substrate for trypsin. Samples of the BBI and DEW were diluted in twofold series in a 96-well plate in quadruplet rows. One hundred μl of porcine trypsin (Sigma) dissolved in 1 mM HCl (Merck) at concentration of 0.0125 mg/ml was added to triplicate samples. As a negative control 1 mM HCl was added to one row of samples. Reagents in the experimental plates were mixed by shaking for 15 min at room temperature. One hundred μl of 6 mM BAPNA (Sigma) was added to each well and incubated for 30 min at 37°C. Fifty μl of 30% acetic acid was added to each well and the absorbance was read at 410 and 690 nm using a POLARstar microplate reader (BMG Labtech). Data were normalized by calculating A410-A690 in Microsoft Excel for each well, and by subtracting the values for corresponding samples without trypsin (1 mM HCl control). Curves were plotted in Excel and EC_50_ values were generated using a linear regression of data from the assay linear range.

### Stability of Immunoglobulins in OraCAb to Simulated Gastric Fluid (SGF)

Simulated gastric fluid, pH 1.2 was prepared according to the US Pharmacopeia and contained 2 g NaCl, 7 ml 37% HCl and 3.2 g porcine pepsin (8000 U) (Sigma) made up to a liter with water for irrigation. OraCAb was evaluated for stability of immunoglobulins to SGF. SGF was mixed with OraCAb formulation in different ratios (10% OraCAb, 20% OraCAb, 30% OraCAb, 40% OraCAb, and 50% OraCAb in SGF). The mixtures were incubated for 2.5 h at 37°C with gentle shaking every 15 min. At the end of the incubation, pH was increased by adding Phosphate Buffered Saline (PBS) to prevent further protein digestion by the pepsin in SGF. Samples were then analyzed by the toxin neutralization assay.

### Toxin Neutralization Assay

Toxin neutralization assays were performed as described previously (Roberts et al., 2012), with modification. Vero cells were seeded at 9 × 10^3^ cells per well in 96-well black microtiter-plates (flat bottom, VWR) in Dulbecco’s Modified Eagle’s Medium (DMEM) supplemented with Penicillin-Streptomycin (100 U/ml and 0.1 mg/ml, respectively; Sigma), Glutamine (2.0 mM; Sigma), HEPES (25 mM Sigma) and 10% Fetal Bovine Serum (Sigma), and incubated for 24 h at 37°C in a humidified incubator under 5% CO*2*. Purified TcdA or TcdB proteins were diluted in DMEM and prepared as 40 ng/ml or 400 pg/ml dilutions, respectively. Suitably diluted oral formulation, anti-TxA4 and anti-TxB4 antibodies were pre-mixed with either TcdA or TcdB in a 96-well culture plate and 100 μl of the pre-mix was transferred to the corresponding wells of the prepared plates with Vero cells. Plates were incubated at 37°C in 5% CO*2* for 48 h. Each dilution was performed in duplicate. Forty μl of Cell-Titer blue stain (Promega) was added to each well and incubated for a further 4 h to determine cell viability. Fluorescence was determined using a POLARstar microplate reader (BMG Labtech) at λ590 nm. The cell survival percentage was calculated in Microsoft Excel and plotted for each concentration using GraphPad Prism. EC_50_ values were calculated using non-linear regression (Sigmoidal fit, 4PL).

### Bacterial Strains

Two *C. difficile* strains were used in this study.

The highly virulent VPI 10463 strain (ribotype 087/toxinotype 0) was obtained from the American Type Culture Collection (ATCC 43255). Spores were prepared from this strain and used in *in vivo* models as previously described (Roberts et al., 2012). The VPI10463 strain is a high producer of both TcdA and TcdB that makes the hamster model of CDI described below a very stringent test for the efficacy of OraCAb.

The epidemic 027 210 strain (BI/NAP1/PCR ribotype 027/toxinotype III) was originally isolated during an outbreak of CDI at the Maine Medical Centre (Portland, ME, United States) in 2005 and was kindly supplied by Dr Robert Owens. Spores were prepared from this strain and used in *in vitro* gut models as previously described (Freeman et al., 2003). The strain used in the *in vitro* studies was chosen as it is an example of an epidemic, so-called ‘hypervirulent’ ribotype 027. Importantly, we have a wealth of previous gut model data using this strain, so we can make meaningful comparisons with previous data. This is particularly helpful with a system such as this gut model which is time consuming and labor intensive, so multiple experimental repeats are not feasible.

### Animal Model for *C. difficile* Infection

**FIGURE 1:**
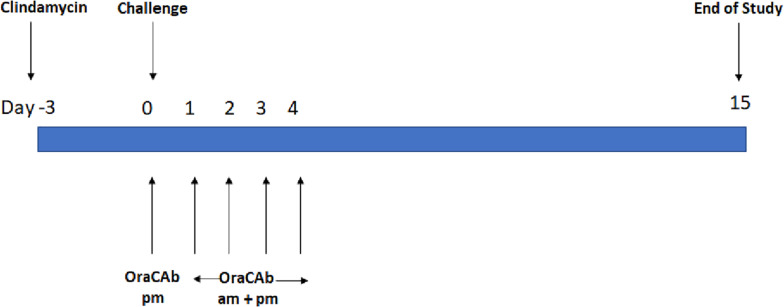
Treatment design of the *in vivo* hamster model of CDI. Each experiment contains sentinel group (x4) that receive clindamycin only and an untreated control group (x10) receiving clindamycin and challenge with *C. difficile*.

The Syrian hamster model was used as described previously (Roberts et al., 2012; Maynard-Smith et al., 2014), using groups of 10 animals (Figure 1). All animals received clindamycin, oro-gastrically (o.g.), on day 0 (Sigma Aldrich; 2.0 mg in 0.2 ml sterile water). On day 3, the animals in the test groups received a *C. difficile* VPI 10463 spore challenge (o.g.; approximately 10^3^ colony forming units in 0.2 ml DMEM). Animals in the treatment groups were administered 0.75 ml of the appropriate OraCAb formulation 9 hrs post-challenge, using 18-gauge 76 mm metal feeding tubes (Linton Instruments). For the following 4 days, these animals received OraCAb treatment twice a day (0.75 ml/dose; 9 h interval between doses). A sentinel group (4 animals) received clindamycin HCl, but no *C. difficile* spore challenge and acted as a cross-infection control for the experiment. Animals were not fasten prior dosing as per the Home Office license under which this experiment was carried out and they were fed *ad libitum* that would ensure that OraCAb encounters similar conditions during gut transit at each dosing.

The animals were weighed daily and monitored 6 times in a 24 h period for symptoms of CDI (diarrhoea, weight loss, lethargy, tender abdomen, activity). Animals in advanced stages of disease were euthanized. When surviving animals were symptomless for 3 days, they continued to be weighed daily and monitored twice daily.

Four different animal model studies were performed. Study A consisted of four test groups of 10 animals; (1) Untreated, (2) Received 150 mg/mL OraCAb formulation containing 3:1 anti-TxB4 IgG (112.5 mg/ml): anti-TxA4 IgG (37.5 mg/ml) ratio with BBI, (3) Received 150 mg/ml OraCAb formulation containing 1:1 anti-TxB4 IgG (75 mg/ml): anti-TxA4 IgG (75 mg/ml) ratio with DEW (International Egg Products UK), (4) Received 150 mg/ml OraCAb formulation containing 3:1 anti-TxB4 IgG: anti-TxA4 IgG ratio with DEW. Study B consisted of three test groups of 10 animals; (1) Untreated, (2) Received 150 mg/ml OraCAb formulation containing 3:1 anti-TxB4 IgG: anti-TxA4 IgG ratio with DEW, (3) Received 150 mg/ml OraCAb formulation containing 3:1 anti-TxB4 IgG: anti-TxA4 IgG ratio without DEW. Study C consisted of four test groups of 10 animals; (1) Untreated, (2) Received 150 mg/mL OraCAb formulation containing 3:1 anti-TxB4 IgG: anti-TxA4 IgG ratio with DEW, (3) Received 150 mg/mL OraCAb formulation containing 1:1 anti-TxB4 IgG: anti-TxA4 IgG ratio with DEW, (4) Received 112.5 mg/mL OraCAb formulation containing anti-TxB4 IgG only with DEW. Study D consisted of four test groups of 10 animals; (1) Untreated, (2) Received 50 mg/ml OraCAb formulation containing 3:1 anti-TxB4 IgG (37.5 mg/ml): anti-TxA4 IgG (12.5 mg/ml) ratio with DEW, (3) Received 100 mg/ml OraCAb formulation containing 3:1 anti-TxB4 IgG (75 mg/ml): anti-TxA4 IgG (25 mg/ml) ratio with DEW, (4) Received 150 mg/ml OraCAb formulation containing 3:1 anti-TxB4 IgG (112.5 mg/ml): anti-TxA4 IgG (37.5 mg/ml) ratio with DEW. The compositions of the formulations are summarized in Table 1.

### *In vitro* Gut Model for *C. difficile* Infection

Gut model studies were performed using a three-stage continuous culture model of the colon. This model is validated against sudden death patients for assessment of the colonic environment and ecosystem (Macfarlane et al., 1998) and has been developed for use with *C. difficile* (Freeman et al., 2003). Three vessels were arranged in a weir cascade with each having the pH controlled to model the proximal (Vessel 1, pH 5.5 ± 0.2), transverse (Vessel 2, pH 6.2 ± 0.2), and distal colon (Vessel 3, pH 6.8 ± 0.2). Vessel 1 had a volume of 280 ml, and vessels 2 and 3 had a volume of 300 ml. Temperature was maintained at 37°C and an anaerobic environment was retained by continuously sparging with oxygen-free nitrogen. To maintain nutrient availability, a nutrient dense medium was added to vessel 1 at a rate of 13.2 ml/h by a peristaltic pump, and any overflow from vessel 3 was collected as waste.

**FIGURE 2:**
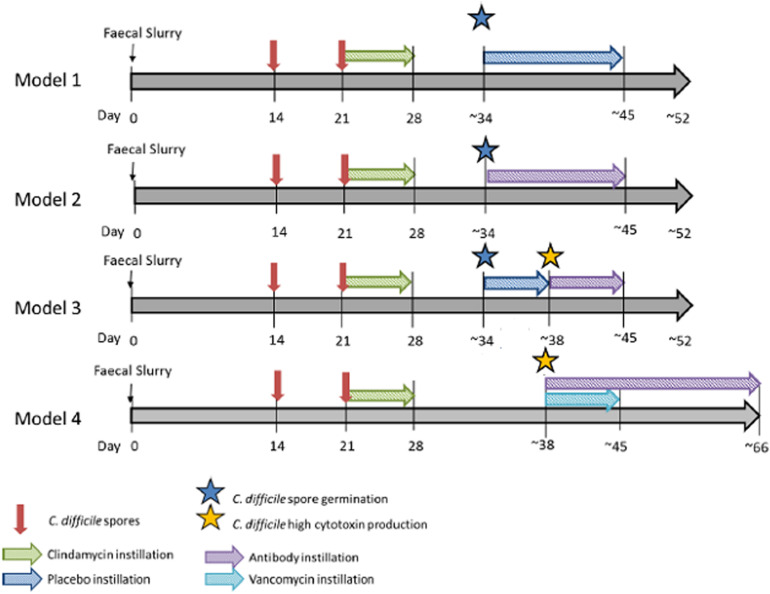
*In vitro* gut model experimental design. Timeline for gut models, including equilibration of microbial community, induction of CDI and addition of placebo, OraCAb antibodies or antibodies + vancomycin.

Four gut model experiments were performed to assess the effect of OraCAb antibodies on *C. difficile*. Models were seeded with stool samples from a minimum of three healthy individuals, aged > 60 years, who had not received antibiotics in the previous 3 months. Stool samples were pooled, and slurries were produced in a 1:10 w/v solution with pre- reduced PBS, equal amounts of slurry were added to each vessel. The models had no further intervention within the first 14 days to allow the bacterial communities to equilibrate and stabilize. At day 14, and day 21, a 1 ml aliquot of *C. difficile* spores (∼7 log_10_ cfu/ml, strain 210) was added to vessel 1 of each model. To induce CDI, vessel 1 of each model was treated with clindamycin (33.9 mg/l, four times daily). It was at this point that the treatments for the models diverge (Figure 2). Model 1; treatment with the placebo (sodium citrate buffer, three times daily, 10 days) once spore germination was detected [defined by an increase in total viable counts (TVCs) compared to spore counts], Model 2; Treatment with 0.18 mg/ml anti-TxA4 IgG + 1.6 mg/ml anti-TxB4 IgG (three times daily, 10 days) once spore germination was detected, Model 3; treatment with placebo once spore germination was detected, then treatment with 0.18 mg/ml anti-TxA4 IgG + 1.6 mg/ml anti-TxB4 IgG (three times daily, 10 days) once high toxin was detected (cell cytotoxicity assay of three relative units), Model 4; Treatment with vancomycin (125 mg/l four times daily, 7 days), and 0.18 mg/ml anti-TxA4 IgG + 1.6 g/l anti-TxB4 IgG (three times daily, ∼35 days), once high toxin was detected.

Gut bacteria, *C. difficile* TVCs and spores were enumerated three times a week during steady state, and daily thereafter using selective agars for facultative and obligate anaerobes as described previously (Chilton et al., 2014a). Samples for cytotoxin detection were taken throughout the experiment and stored at 4°C as described previously (Chilton et al., 2014a). Samples for antimicrobial bioassay were taken post-antibiotic exposure and stored at −20°C prior to processing as described previously (Chilton et al., 2014b).

### Statistics

Hamster survival data at days 5 and 15 within each plot were analyzed using the Gehan-Breslow test followed by the Holm–Sidak method for pairwise comparison of the groups. Differences between groups were considered statistically significant when *p* < 0.05.

## Results

### TcdA and TcdB Neutralization *in vitro* by OraCAb Formulation

The amount of OraCAb ovine anti-TxA4 IgG and OraCAb anti-ovine TxB4 IgG required to neutralize 1.0 ng of TcdA or TcdB, respectively, was calculated using a Vero cell-based toxin neutralization assay. To neutralize 1 ng of TcdA, 0.156 μg of anti-TxA4 IgG were sufficient. For neutralization of 1 ng of TcdB 8 μg of anti-TxB4 IgG were required. The lower neutralizing capacity of antibodies to TxB4 was taken into account in the design of the protocols used in the hamster model and the *in vitro* gut model. Previously, we determined that antibodies to both toxins were required for protection in the hamster model when the antibodies were administered systemically (Roberts et al., 2012). However, others have shown that systemic administration of antibodies against TcdA and TcdB do not improve the efficacy of treatment of CDI compared to administration of anti-TcdB only in piglets (Steele et al., 2013) and humans (Lee et al., 2017). Considering the specificity of different CDI models, the *in vivo* studies described here used formulations with anti-TxB4:anti-TxA4 ratios up to 3:1, while for the treatment of CDI in the *in vitro* human gut model we used a ratio of approximately 9:1. Anti-TxA4 was not abolished completely in the human gut model as in this study antibodies were administered orally to act directly at the site of infection, thus in an *in vivo* setup anti-TxA4 would protect gut epithelium from further damage and prevent infiltration of antibodies in the circulation.

### Protection of Antibodies in the Formulation From Enzymatic Digestion in the GI Tract

**FIGURE 3:**
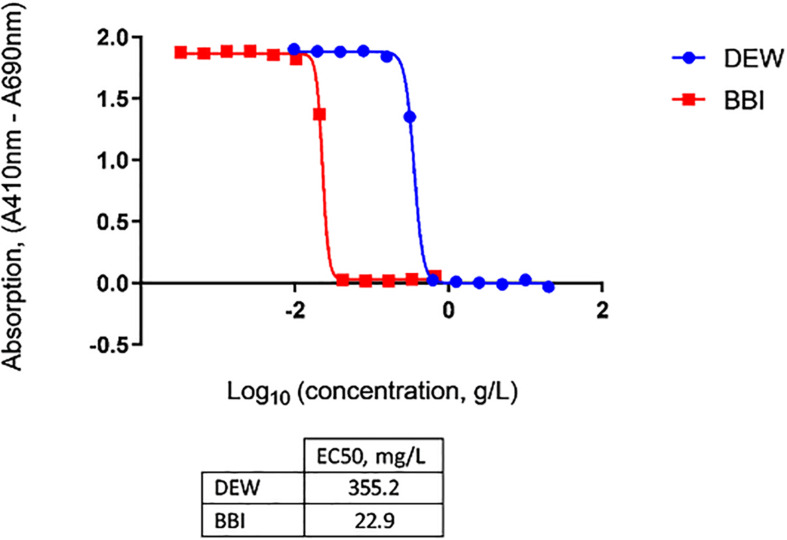
Inhibition of human trypsin with DEW and purified BBI determined by *N*α-Benzoyl-DL-Arginine-*p*-Nitroanilide (BAPNA) colorimetric assay. Concentrations of DEW and BBI that gave half-maximal inhibition of human trypsin (EC_50_) are given in the table.

To enable effective neutralization by OraCAb, the antibodies in the formulation must be delivered intact to the large intestine. Trypsin is the major proteolytic enzyme of the small intestine. DEW and BBI, the latter which can be purified from Lima beans, are two options that can be used as inhibitors of trypsin in OraCAb formulation to protect the integrity of the antibodies. Thus, we investigated the inhibition of human trypsin *in vitro* by either DEW or BBI. The BBI inhibitor was more potent, with approximately 15-fold less BBI being required, compared to DEW, to inhibit the same amount of human trypsin (Figure 3). This data together with available information on concentration of trypsin in human GI tract (Borgstrom et al., 1957) were used to inform levels of the two inhibitors to be included in the OraCAb formulations used in the *in vivo* experiments.

### Protection of Antibodies in the Formulation From Acid pH

**FIGURE 4:**
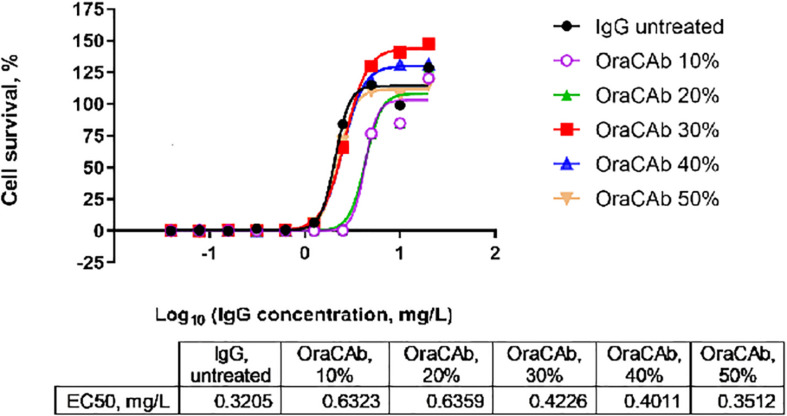
Stability of IgG (anti-TxA4) in OraCAb formulation in SGF. OraCAb was incubated for 2.5 h in SGF at different v/v ratios and full protection of the IgG was found when the volume of OraCAb was 30% (3/10 ratio) or higher compared with the volume of the SGF. Around 50% of the IgG was found inactivated/degraded in SGF containing 10 or 20% OraCAb. Concentrations of IgG in OraCAb that give half maximum inhibition of TcdA in a cell-based toxin neutralization assay (EC_50_) are given in the table. The calculations were based on the concentration of IgG prior to mixing the formulations with SGF.

The low pH in human gastric fluid (1.5–3.5) and the presence of pepsin, would inactivate the proteins in the OraCAb formulation by affecting their conformation and integrity. OraCAb formulation contains antacids (magnesium hydroxide and aluminum hydroxide), which have been used widely in medicines for the treatment of dyspepsia, to protect the antibodies and protease inhibitors from inactivation in the stomach. The presence of antacids will also raise the stomach pH leading to inactivation of pepsin. The antacids neutralize the pH in the stomach once the OraCAb is taken. The aluminum hydroxide gel is also needed to maintain the higher pH while the drug is present within the stomach and additional gastric fluids are released. Antacid concentrations in OraCAb provide a similar daily intake as that from dyspepsia medications. We studied the dose of OraCAb that needed to be administered orally to ensure full protection of the IgG in a simulated gastric fluid (SGF). The data (Figure 4) show that OraCAb mixed with the SGF at 3/10 v/v ratio was sufficient to fully preserve the activity of the IgG. An example of the protection of anti-TxA4 IgG in OraCAb from SGF is given in Figure 4.

### A Trypsin Inhibitor Appears to Preserve OraCAb Activity *in vivo*

**FIGURE 5:**
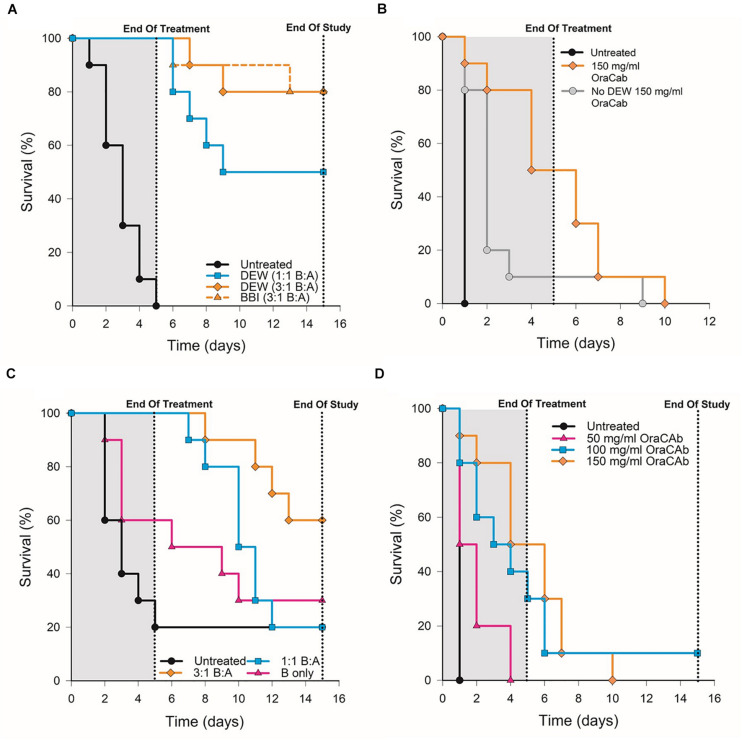
Survival rates of hamsters after *C. difficile* challenge. Hamsters were challenged with *C. difficile* spores via the oral route on day 0, placed on one of the oral therapeutic regimes until the end of day 4 and monitored until day 15. An untreated control group, receiving challenge only, was included for each experiment. **(A)** Groups were treated with OraCAb containing different ratios of anti-TxB4: anti-TxA4 antibodies with either BBI or DEW as trypsin inhibitors. **(B)** Groups were treated using OraCAb formulated with antibody concentration of 150 mg/ml at a 3:1 anti-TxB4: anti-TxA4 ratio in the presence or absence of DEW. **(C)** Groups were treated with OraCAb containing different ratios of anti-TxB4: anti-TxA4 antibodies. The antibodies in OraCAb that contained anti-TxB4 IgG only were at the same concentration as anti-TxB4 antibodies in the 3:1 anti-TxB4: anti-TxA4 formulation. All OraCAb formulations contained DEW. **(D)** Groups were treated with OraCAb containing DEW and three different antibody concentrations (50, 100, or 150 mg/ml) at a fixed 3:1 anti-TxB4: anti-TxA4 ratio. The table shows the outcome of the statistical analyses of the data.

To determine whether OraCAb toxin neutralization can prevent CDI *in vivo*, we used the Syrian hamster model. Animals were given clindamycin orally 3 days before challenge and then challenged with *C. difficile* spores via the oral route on day 0. An untreated group acted as controls and a sentinel group that received clindamycin, but no challenge, was included to check for cross-contamination. Test groups contained 10 animals and sentinel groups consisted of four animals. The animals were then placed on one of the oral therapeutic regimes until the end of day 4 (Table 1) and monitored until day 15. Statistical analyses were performed on day 5 (end of treatment) and day 15 (end of the study). Survival results for each study are shown in Figure 5 and Table 2.

**TABLE 2:** *P*-values calculated for different treatments in the *in vivo* model of CDI.

**Study**	**Comparison**	***P*-value end of study**	***P*-value end of treatment**
A	OraCAb DEW 3:1 B:A vs. untreated	<0.001	<0.001
	OraCAb BBI 3:1 B:A vs. untreated	<0.001	<0.001
	OraCAb DEW 1:1 B:A vs. untreated	<0.001	<0.001
	OraCAb DEW 3:1 B:A vs. OraCAb BBI 3:1 B:A	1.0	1.0
B	OraCAb DEW 3:1 B:A vs. untreated	0.008	<0.001
	OraCAb No DEW 3:1 B:A vs. untreated	0.003	0.001
	OraCAb DEW 3:1 B:A vs. OraCAb No DEW 3:1 B:A	0.021	0.014
C	OraCAb DEW 3:1 B:A vs. untreated	0.018	0.003
	OraCAb DEW 1:1 B:A vs. untreated	0.049	0.002
	OraCAb DEW anti-TxB4 only vs. untreated	0.30	0.18
D	OraCAb 50 mg/ml IgG vs. untreated	0.035	0.004
	OraCAb 100 mg/ml IgG vs. untreated	0.002	0.002
	OraCAb 150 mg/ml IgG vs. untreated	0.008	<0.001
	OraCAb 50 mg/ml IgG vs. OraCAb 150 mg/ml IgG	0.016	0.016
	OraCAb 150 mg/ml IgG vs. OraCAb 100 mg/ml IgG	0.3	0.3

We investigated the effects of inclusion of two different protease inhibitors in the OraCAb formulation, BBI (10 mg/ml) and DEW (60 mg/ml) (Figure 5). In study A, no significant difference was observed between groups given formulations containing DEW inhibitor and those receiving a formulation containing BBI trypsin inhibitor (*p* = 1.0, Figure 5A), indicating the benefit of these two inhibitors to be comparable in the hamster model. In study B, disease progression was rapid with no untreated animals surviving to the second day post challenge. On day four the survival rate in the group treated with OraCAb containing DEW was 50%, in the absence of protease inhibitor it was 10% (Figure 5B). By day ten there were no survivors in either group. In this study there was a significant difference between the survival of animals given OraCAb containing DEW and those treated with OraCAb where DEW was omitted (*p* = 0.021) and a clinical benefit of the addition of DEW can be observed during the treatment period.

### OraCAb Formulations Containing Antibodies to Both TcdA and TcdB Protect Hamsters From CDI

During the production and assessment of the ovine antibodies to TxA4 and TxB4, we noted that the toxin neutralizing capacity of ovine anti-TxB4 IgG was lower than that of ovine anti-TxA4 IgG. In addition, in previous studies we determined that antibodies to both toxins were required for protection in the hamster model when the antibodies were administered systemically (Roberts et al., 2012). Therefore, we assessed the efficacy of different OraCAb formulations containing different ratios of anti-TxB4: anti-TxA4 IgG, as well as a formulation containing anti-TxB4 IgG only.

All animals that received an OraCAb formulation containing both anti-TxB4 and anti-TxA4 antibodies had a significantly higher survival rate compared to untreated animals, whereas those receiving anti-TxB4 IgG only did not (Figure 5C). In study C (Figure 5C), 80% of the untreated animals succumbed to CDI 5 days post-challenge. During this time interval, all animals treated with OraCAb containing antibodies to both toxins remained healthy. However, 40% of the group receiving anti-TxB4 IgG only had succumbed to CDI. There was a significant difference between the survival rates of animals receiving 3:1 anti-TxB4: anti-TxA4 IgG and 1:1 anti-TxB4: anti-TxA4 IgG compared to the control group (*p* = 0.018 and 0.049, respectively). Animals receiving OraCAb containing antibodies that neutralize TcdB only did not experience a statistically meaningful clinical benefit over untreated animals (*p* = 0.30). In study A (Figure 5A), all untreated animals succumbed to CDI by day 5 post-challenge, in contrast to animals receiving OraCAb, where all animals were protected from CDI during this period. Fifty percent of the animals receiving OraCAb with 1:1 anti-TxB4 to anti-TxA4 IgG ratio and 80% of animals receiving OraCAb with 3:1 anti-TxB4 to anti-TxA4 IgG ratio were protected and survived to the end of the study (*p* < 0.0001 compared to the untreated group). All OraCAb formulations produced a significant clinical benefit relative to the untreated control group. OraCAb, containing antibodies that neutralize both TcdA and TcdB, appears to be protective in the hamster model of CDI. The presence of antibodies to TcdA in the gut appears to give increased efficacy of OraCAb in the hamster model of CDI. A direct ELISA for detection of infiltrated ovine IgGs in the sera from hamsters with CDI and treated with OraCAb was developed (see Supplementary Material). No presence of ovine IgG was detected in any of the tested 15 sera (Supplementary Figure S2). Although the sera samples were collected 10 days after the final OraCAb treatment, the high sensitivity of the ELISA and the long half-life of ovine IgGs (more than 10 days, Watson, 1992) strongly support the conclusion that there was no significant systemic infiltration of ovine IgGs in hamsters with CDI.

### OraCAb Formulations Containing 3:1 Ratio of Anti-TxB4 to Anti-TxA4 IgG May Be Superior to Those Containing 1:1 Ratios

For both studies A and C (Figures 5A,C), a higher survival rate was observed for animals receiving the 3:1 anti-TxB4 to anti-TxA4 IgG OraCAb formulation compared with those receiving the 1:1 antibody formulation. In study A, 80% of the animals receiving the 3:1 formulation survived to the end of the study compared to 50% given the 1:1 ratio OraCAb. For study C, percentage survival was 60 and 20% for the 3:1 ratio and the 1:1 ratio, respectively. Although survival data between the two antibody ratios did not reach statistical significance in either study, the data generated from both studies indicates a better survival outcome and clinical benefit in the hamster model when animals were treated with the OraCAb formulation with a ratio of 3:1 anti-TxB4 to anti-TxA4 IgG. The statistical method used to assess significance between different treatment groups is based on the area under the survival curves. Thus, although a difference in the survival rate of these two groups was observed after the end of the treatment, significance between these two groups was not achieved most likely due to their comparable outcome during the treatment period.

### OraCAb Administration Protects Hamsters in a Dose-Dependent Manner

We investigated the dose dependency of OraCAb using three different concentrations of antibody in a 3:1 ratio of anti-TxB4:anti-TxA4 IgG. In study D, CDI progression was rapid with no untreated animals surviving to the second day post challenge (Figure 5D). Compared to the untreated group, all OraCAb formulations provided a significant increase in survival rate (50 mg/ml *p* = 0.035, 100 mg/ml *p* = 0.002, 150 mg/ml *p* < 0.0005). The 150 mg/ml antibody OraCAb provided significantly better clinical benefit compared to the 50 mg/ml antibody OraCAb formulation (*p* = 0.016). No statistically meaningful differences were observed between treatment groups receiving 100 mg/ml and 150 mg/ml antibody OraCAb formulations (*p* = 0.30). The data suggest that OraCAb containing greater than 50 mg/ml IgG is required for protection from CDI in the hamster model.

### OraCAb Antibody Instillation Prevents Toxin Detection in an *in vitro* Gut Model of CDI

A well validated and clinically reflective *in vitro* gut model of CDI was used to investigate the efficacy of OraCAb instillation in the presence of the normal human microbiota. The models were populated with a pooled fecal slurry and left to equilibrate before simulated CDI was induced using clindamycin. Models were treated with either (A) placebo (sodium citrate saline buffer), (B) OraCAb antibodies alone, (C) placebo followed by OraCAb antibodies (D) OraCAb antibodies alongside standard of care (vancomycin). The OraCAb antibodies were formulated as 9:1 anti-TxB4 IgG: anti-TxA4 IgG in a sodium citrate saline buffer. One of the benefits of using an *in vitro* system such as the gut model in pre-clinical evaluation is that longitudinal trends (such as *C. difficile* spore germination and toxin production) can be followed in a way that is not possible clinically. In models A and B it was specifically chosen to start instillation at the point of spore germination in order to assess the impact of OraCAb as an early intervention (to reflect the use of OraCAb as prophylaxis, e.g., in prevention of recurrence). In Model C, it was instilled from toxin detection (simulating the point at which a patient was symptomatic), and in Model D alongside the ‘standard of care’ vancomycin. Thus we were able to gain data on the potential efficacy of OraCAb administered in a variety of clinical scenarios.

**FIGURE 6:**
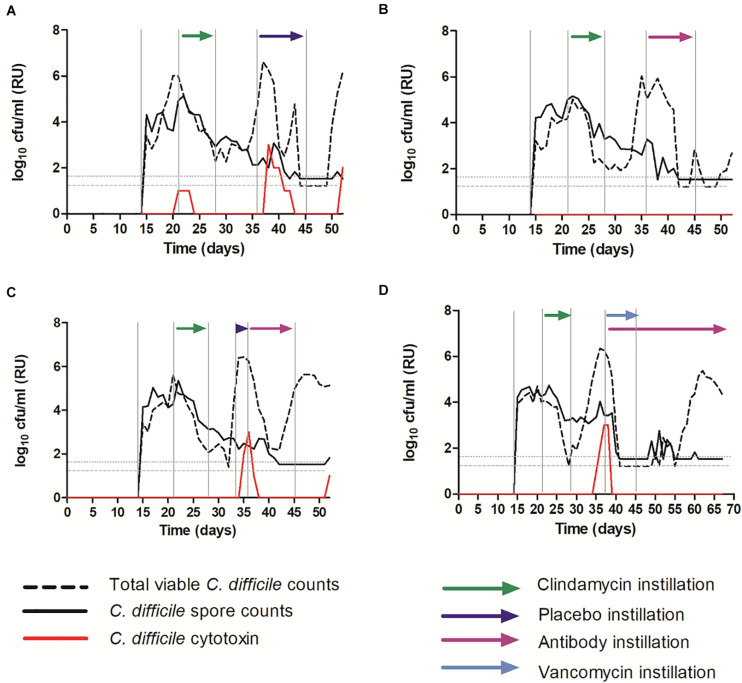
Mean *C. difficile* total viable counts (log_10_ cfu/ml), spore counts (log_10_ cfu/ml) and cytotoxin titer [relative units (RU)] in vessel three of the *in vitro* gut model. **(A)** – Model 1 (placebo); **(B)** – Model 2 (antibodies); **(C)** – Model 3 (placebo + antibodies); **(D)** – Model 4 (vancomycin + antibodies). The limit of detection is 1.22 log_10_ cfu/ml for total viable counts and 1.52 log_10_ cfu/ml for spore counts; horizontal dotted lines.

Clindamycin exposure induced simulated CDI in all models (Figure 6). Simulated CDI is defined by an increase in TVCs over spore counts (indicating *C. difficile* spore germination and vegetative cell proliferation), with concomitant high cytotoxin detection. Instillation of placebo did not prevent *C. difficile* vegetative growth or toxin detection (Figure 6A). TVCs peaked at ∼6.5 log_10_ cfu/ml (spore counts ∼2 log_10_ cfu/ml) and a peak toxin titer of 3 relative units was observed. Both *C. difficile* vegetative growth and toxin production reduced, before increasing again at the end of the experiment. Instillation of OraCAb antibodies at the first sign of *C. difficile* spore germination (increasing TVC over spore counts) did not affect *C. difficile* vegetative growth (TVCs peaked at ∼6 log_10_ cfu/ml with spores ∼2.5 log_10_ cfu/ml), but completely prevented the detection of *C. difficile* cytotoxin (Figure 6B). Instillation of placebo at the first sign of spore germination followed by instillation of OraCAb antibodies at simulated CDI (high toxin detection) led to a rapid decrease in detectable toxin (2 days after peak toxin compared to 5 days after peak toxin in the placebo treated model). No further toxin was detected during OraCAb antibody instillation, apart from a low level titer of 1 on the final day of experiment. This was despite a second increase in TVC counts compared to spores, indicating prolonged vegetative cell proliferation (Figure 6C).

### OraCAb Antibody Instillation Alongside Standard of Care (Vancomycin) Prevents Simulated Recurrent CDI in an *in vitro* Gut Model of CDI

Instillation of OraCAb antibodies alongside vancomycin caused rapid reduction in *C. difficile* vegetative cells and cytotoxin levels, as has been observed previously when vancomycin is used to treat simulated CDI in the gut models (Freeman et al., 2005; Baines et al., 2008; Chilton et al., 2012, 2014b; Crowther et al., 2015). No further toxin was detected throughout the experiment (Figure 6D). Importantly, in model 4 (Figure 6D), recurrent *C. difficile* vegetative cell growth was observed post vancomycin instillation. It would be expected that this would be accompanied by cytotoxin production (simulated recurrent disease) as has been observed previously (Chilton et al., 2012, 2014b; Crowther et al., 2015), however, with the installation of the OraCAb antibodies, no toxin was detected.

### OraCAb Antibody Instillation Did Not Affect Gut Microbiota Populations in an *in vitro* Gut Model of CDI

**FIGURE 7:**
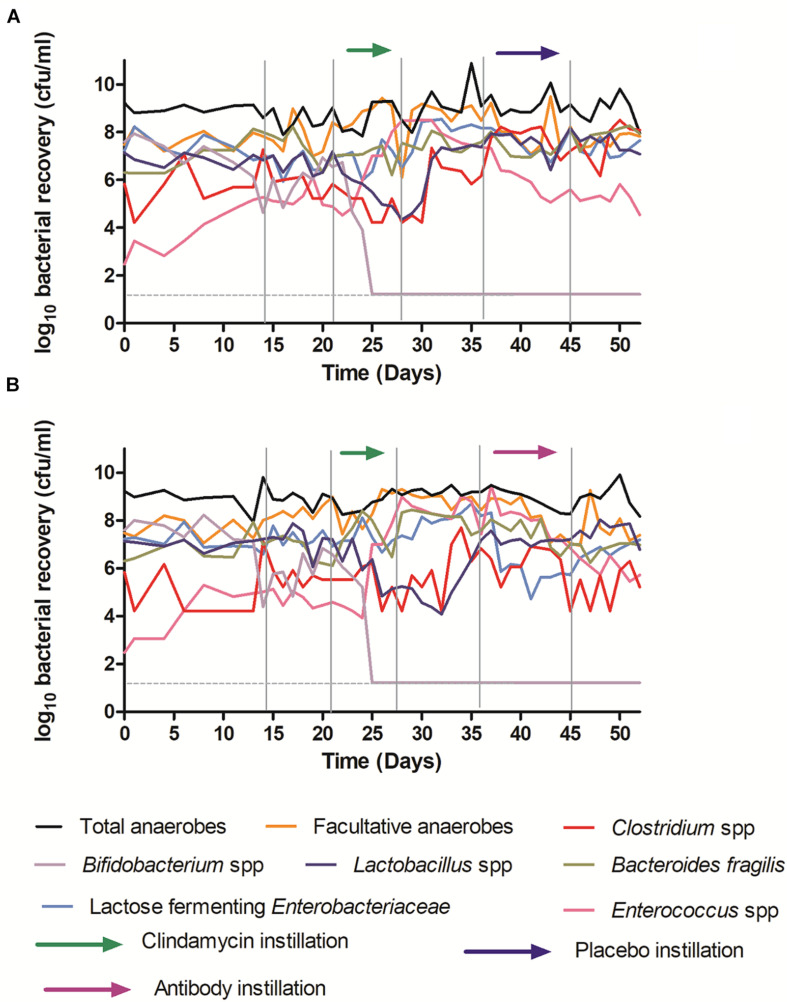
Mean viable counts of selected microbiota communities in vessel three of the *in vitro* gut model. **(A)** – Model 1 (placebo); **(B)** – Model 2 (antibodies). The limit of detection is 1.22 log_10_ cfu/ml; horizontal dotted line.

Instillation of antibodies did not affect microbiota populations, the dynamics of which were similar in all experiments and to those from previously reported *in vitro* gut model studies (Freeman et al., 2005; Baines et al., 2008; Chilton et al., 2012, 2014a; Crowther et al., 2015). The results from models 1 and 2, receiving placebo and antibody are shown here (Figure 7).

Although some variation was observed between individual models, clindamycin instillation had similar effects to those reported previously in the *in vitro* gut model of CDI, causing declines in *Bifidobacterium* species (∼6 log_10_ cfu/ml to below the limit of detection), and *Lactobacillus* populations (∼2–4 log_10_ cfu/ml), and increases in lactose fermenting *Enterobacteriaceae* (∼2 log_10_ cfu/ml) and *Enterococcus* species (∼4 log_10_ cfu/ml). Recovery following clindamycin exposure was similar regardless of whether models were exposed to antibodies or placebo. These data provide an important indication that the effects of OraCAb on humanized gut microbiota is minimal.

These results highlight the protective capacity provided by an orally delivered ovine polyclonal antibody formulation designed to neutralize both TcdA and TcdB. The formulation protects hamsters from CDI and the antibodies have been shown to neutralize TcdA and TcdB in the human gut model. The antibodies can be used alongside standard of care antibiotics and they do not have a deleterious effect on the microbial flora.

## Discussion

CDI remains a problem within healthcare systems of the developed (Lessa et al., 2015) and the developing world (Chaudhry et al., 2017). It is interesting that at present approximately 60% of CDI cases are defined as ‘community-acquired,’ but this leads to further cases needing hospitalization (Ofori et al., 2018; Public Health England, 2019). Even with effective therapy, recurrent infection is common, ranging from 20% after initial infection to 60% after multiple prior recurrences (McFarland et al., 1999), suggesting that antibiotic treatment has failed to eradicate *C. difficile* and/or exacerbated the dysbiosis that initially allowed the bacterium to proliferate and produce toxin. Therefore, additional therapeutic options are required for use when antibiotic treatment alone fails (Jawa and Mercer, 2012). For example, a combined standard of care antibiotic and systemically administered bezlotoxumab, a MAb against TcdB, has been proved to be clinically effective in the prevention of recurrent CDI (Wilcox et al., 2017).

Previously we described development of a therapeutic for CDI based on the administration of ovine polyclonal antibodies to novel, non-toxic recombinant antigens, TxA4 and TxB4, based on TcdA and TcdB. This was the first use of ovine serum antibodies in the treatment of CDI. These antibodies potently neutralize TcdA and TcdB in both *in vitro* cell assays and in an *in vivo* hamster model for CDI (Maynard-Smith et al., 2014). In the present study we show that these ovine antibodies can also be formulated for oral delivery and can act as an effective therapeutic for CDI. We also show in an *in vitro* human gut model that the antibodies prevent the detection of toxin activity during simulated recurrent CDI. Unlike antibiotics, OraCAb antibodies do not have a deleterious effect on the human gut flora. This is the first study to describe the use of orally delivered ovine polyclonal IgG for the treatment of CDI.

To our knowledge, this is the first use of an *in vitro* gut model seeded with a human fecal inoculum to test the effects of oral antibodies for *C. difficile* infection. Treatment with clindamycin and vancomycin, resulted in a loss of colonization resistance and led to CDI, as previously identified in gut model studies (Chilton et al., 2014a; Crowther et al., 2015). OraCAb repeatedly neutralized *C. difficile* cytotoxin, Figure 6C demonstrating that within 48 h the toxin produced was neutralized from a titer of 3 RU to undetectable. Additionally, consistent treatment with OraCAb, prevented the detection of *C. difficile* cytotoxin for greater than 48 h (Figures 5B–D). Of note, although OraCAb prevented the detection of cytotoxin, it did not prevent the growth and spore germination of *C. difficile* itself. OraCAb has been developed to reduce the toxin burden in the gut and thus it was not expected to have anti-*C. difficile* properties. As CDI is a toxin mediated disease (Lyerly et al., 1985), these results suggest that OraCAb would be beneficial, as a potential prophylaxic, alongside standard of care treatments for CDI.

Passive immunization, using both polyclonal (mostly of bovine colostrum and chicken egg origin) and MAbs, to protect against CDI has been studied extensively in animal models (Kink and Williams, 1998; Roberts et al., 2012; Maynard-Smith et al., 2014; Sponseller et al., 2015; Pizarro-Guajardo et al., 2017). Hyperimmune ovine serum, unlike bovine colostrum, can be continually supplied and requires only immunization boosts at certain time intervals. Polyclonal antibodies have several advantages over MAbs. Firstly, they are cheaper to produce, and secondly, they bind to several epitopes on their antigen, increasing the neutralizing efficacy of the therapeutic against sequence variants, such as those observed in TcdA and TcdB of *C. difficile*. Recently, Cole and co-workers demonstrated that polyclonal sera to both TcdA and TcdB when tested in an *in vitro* toxin neutralization assay, displayed greater activity against the toxins than equivalent concentrations of individual MAbs (Cole et al., 2019). Animal-derived polyclonal antibodies have the disadvantage of inducing an immune response in the recipient when delivered systemically and thus the number of doses will be limited. In severe disease, where therapeutic options have been exhausted, one or two doses may be all that is required to alleviate symptoms. Recently, bezlotoxumab, a MAb-based therapy, has been licensed for use in the prevention of recurrent CDI (Wilcox et al., 2017). However, CDI is an infection of the large intestine where the virulence factors act on the intestinal epithelium and therefore the oral delivery of an immunotherapeutic at the site of toxin production and action would be advantageous. Oral administration would be the preferable route for non-severe and/or recurrent CDI allowing repeated treatment to take place due to lack of adverse humoral responses in circulation as indicated by clinical trials with orally delivered polyclonal antibodies against TNFα (AVX-470) for treatment of ulcerative colitis (Harris et al., 2016), a disease which is also associated with severe colon inflammation. Zhang et al. (2015) have argued that the requirement for epithelial disruption to allow transport of systemic antibodies to the site of infection (i.e., the gut lumen) is actually an advantage as it provides a constant source of antibodies over time to defend against future infections. Indeed, this is correct in severe CDI when systemic administration of antibodies against *C. difficile* toxins would have no alternative.

There have been a few clinical trials in which bovine antibodies have been administered orally in healthy or challenged individuals (Jasion and Burnett, 2015). Data from these trials show survival of orally administered immunoglobulins through the digestive tract in humans to be between 0.01 and 20%. These observations along with our data that addition of a protease inhibitor to the formulation led to significant improvement in survival of animals during the treatment (Figure 5B) clearly demonstrate the need for protection of immunoglobulins from digestion and/or inactivation in the GI tract. The OraCAb formulation that we report here protects the proteins in this formulation from degradation/inactivation by low pH in gastric fluid and from proteases during transit from oral administration to the large intestine (Figures 2, 3). Two protease inhibitors were investigated for inclusion in OraCAb. BBI is a highly purified trypsin and chymotrypsin inhibitor from Lima beans. It can pass through the stomach and small intestine without major degradation (Clemente et al., 2011). DEW contains several protease inhibitors (Saxena and Tayyab, 1997). It is known that some proteolytic inhibitors have a feedback control of proteolytic enzyme secretion in humans (Liener et al., 1986) and also in animals, e.g., rats (Geratz and Hurt, 1970). Some trypsin/chymotrypsin inhibitors stimulate secretion of proteolytic enzymes into small intestines (e.g., Bowman-Birk Inhibitor, 4,4′-diamino-diphenylamine), however, others like *p*-aminobenzamidine does not seem to have significant effect on secretion of trypsin and chymotrypsin in small intestine in rats (Geratz and Hurt, 1970). DEW has not been tested for effect on secretion of proteases in small intestines. However, the quantity of trypsin/chymotrypsin inhibitors in OraCAb has been calculated based on data for concentrations of these two enzymes in human duodenum during digestion of standard meal (Worning and Müllertz, 1966) with an excess to allow for possible increased secretion of these proteases. Our *in vivo* data (Figure 5A) showed that the 2.5-fold higher potency of BBI in the formulation compared to that of DEW did not make significant difference in survival of the hamsters. The fact that increasing the potency of trypsin/chymotrypsin inhibitors with a feedback control did not affect the survival rates demonstrate that the concentration of 60 mg/ml DEW was sufficient to ensure protection from proteolytic enzymes in the hamster model of CDI even if a similar feedback control in release of proteolytic enzymes takes place in presence of DEW. Thus, subsequent experiments were performed with OraCAb containing DEW which considerably reduces the manufacture cost of OraCAb.

Over recent years there has been much conjecture over the roles of TcdA and TcdB in CDI. Laboratory-derived TcdA and TcdB gene knock-out mutants, when tested in the gold standard hamster model, have given conflicting results. Using survival data, Lyras et al. (2009) proposed that TcdB was the key virulence factor. However, Kuehne et al. (2010, 2014) and Carter et al. (2015) have both demonstrated that either TcdA or TcdB can cause fulminant disease in the hamster infection model. Furthermore, in previous studies, we have shown that antibodies which neutralize specifically either TcdA or TcdB are both required to provide protection against CDI in the hamster when given systemically. *In vitro* assays demonstrated no neutralization of TcdA by anti-TcdB antibodies and *vice versa* (Roberts et al., 2012; Maynard-Smith et al., 2014). In this study we addressed if both antibodies are required for treatment of CDI in a hamster model when the therapy is given orally, and neutralizing antibodies are delivered directly to the site of infection. Our data showed that antibodies against TcdB alone did not significantly protect from CDI (Figure 5C). This finding suggests that TcdA is important in the pathogenesis of CDI, at least in the hamster model, when a highly toxigenic challenge strain is employed. Apart from their cytotoxic effects on the epithelium of the large intestine, TcdA and TcdB have been shown to be pro-inflammatory by eliciting cytokine release, with TcdA causing a greater pro-inflammatory reaction than TcdB (Yu et al., 2017). Therefore, the neutralization of both toxins might provide better protection of the gut epithelium. The strong inflammatory response induced by TcdA (and TcdB) triggers the release of inflammatory mediators from intestinal epithelial cells which leads to intestinal barrier dysfunction (Hansen et al., 2013; Yu et al., 2017). Thus, TcdA may play a pivotal role in the toxicity of TcdB and the severity of CDI by facilitating systemic entry of both toxins. Neutralization of both toxins at the site of their production might reduce gut damage, decreasing extra-intestinal transit of toxins and systemic effects of severe CDI. Kink and Williams (Kink and Williams, 1998), using orally delivered antibodies derived from eggs, showed that antibodies to both toxins were required to prevent mortality in the hamster model, supporting the results obtained in this study. However, Hutton et al. (2017) suggest that the oral administration of bovine hyperimmune colostrum, containing neutralizing antibodies to TcdB only, is effective in the treatment or prevention of CDI. There were several differences between the current study and the studies undertaken by Hutton and colleagues. For example, in the latter study, the mouse model of CDI was employed and run for 4 days post-challenge during which period mice were given hyper-immune bovine colostrum as the antibody source. A ribotype 027 *C. difficile* strain was used to challenge the mice. In our study, the hamster model was used and run for 16 days post-infection during which purified ovine antibodies against TcdA and TcdB were given for the first 4 days post-challenge only. The highly toxigenic ATCC 43255 strain of *C. difficile* produces high concentrations of TcdA and TcdB *in vitro* (Merrigan et al., 2010). The strain described by Merrigan et al. (2010) and the strain used in these studies was obtained from the same original source. With all these differences in methodology, it is unwise to compare the studies in detail. However, both studies support the hypothesis that toxin neutralizing antibodies can be delivered orally and prevent CDI in an animal model. It should also be noted that recently a variant strain of *C. difficile*, producing only TcdA was isolated from humans (Marvaud et al., 2019), suggesting that antibodies that neutralize TcdA might be beneficial in CDI immune therapeutics.

## Conclusion

OraCAb demonstrated potential to prevent CDI, induced by a highly toxigenic *C. difficile* strain, in the hamster model. A 3:1 ratio of anti-TxB4 IgG to anti-TxA4 IgG with a total IgG concentration of greater than 50 mg/ml IgG was required for protection of hamsters. In addition, using the *in vitro* gut model, it was proposed that OraCAb antibodies might be beneficial when used alongside vancomycin to prevent simulated recurrent CDI. It is important to note that this *in vitro* model is limited to the microbiota of the gut, and cannot model some important factors in CDI, such has host immune response. It is likely that a treatment that prevents toxin (the disease causing agent) from acting on the gut mucosa in the first instance could allow (a) recovery of the microbiota to reduce further *C. difficile* growth (b) action of the immune system to reduce the burden of *C. difficile*. Clearly, clinical trials are required to answer such issues surrounding the capacity of orally administered antibodies to interrupt CDI/gut inflammation. With the pre-clinical development of OraCAb completed, the formulation needs to undergo a Phase1b/2a clinical trial where will be tested for its safety and efficacy. The pre-clinical results presented here could be used as guidance for the design of antibody ratio and concentrations in OraCAb that will be used in clinical trials and subsequent product manufacture.

## Data Availability Statement

The raw data supporting the conclusions of this article will be made available by the authors, without undue reservation.

## Ethics Statement

The studies involving human participants were reviewed and approved by the University of Leeds School of Medicine Research Ethics Committee (Ref: MREC15-070), Leeds, United Kingdom. The patients/participants provided their informed consent to participate in this study. The animal study was reviewed and approved by Ethical Review Process of Public Health England (PHE), Porton, Salisbury, United Kingdom and the Home Office, United Kingdom.

## Author Contributions

AR and CS conceived and designed the experiments performed at PHE. HH, CC, AB, and MW designed the *in vitro* gut model experiments. IM, DE, EC, and WS conducted the *in vitro* gut model and analyzed samples taken from the models. RD, JG, and OP developed the OraCAb formulation and developed and validated the toxin neutralization assay and BAPNA assay for activity of trypsin inhibitors. MS developed the purification protocol for the Bowman–Birk Inhibitor and helped with preparation of samples for the *in vivo* studies. AR also prepared the samples for use in these studies. AR, HH, CC, and RD wrote the manuscript. All authors discussed the results and commented on the manuscript.

## Conflict of Interest

RD, JG, and OP were employed by the company MicroPharm Ltd. AR and CS hold patent WO 2011/067616, “Therapies for preventing or suppressing *Clostridium difficile* infection.” AR, CS, and MS hold patent WO 2012/046061, “*Clostridium difficile* antigens.” The remaining authors declare that the research was conducted in the absence of any commercial or financial relationships that could be construed as a potential conflict of interest.
