# Supplementary material for: A Novel, Orally Delivered Antibody Therapy and Its Potential to Prevent Clostridioides difficile Infection in Pre-clinical Models
Source: Front Microbiol. 2020 Sep 22;11:578903. doi: 10.3389/fmicb.2020.578903 (PMC7537341; doi:10.3389/fmicb.2020.578903)
Supplement: Supplementary file 1 [file Data_Sheet_1.docx]

# 1 Supplementary Material

# 1.1 Detailed purification protocol for Bowman-Birk Inhibitor (BBI)

Lima beans (350 g) were dry blended to produce a rough flour and suspended in ethanol (700 ml) with stirring at room temperature for 2 hours. The bean flour was harvested by filtration, washed with an additional volume (350 ml) of ethanol, air dried and stored at -20°C. Frozen defatted bean flour was resuspended in two volumes (v/w) of 1% acetic acid pH 4 and wet blended for two minutes to produce a fine resuspension. A silicone polymer-based antifoam was added and the volume adjusted to 3.5 L with additional 1% acetic acid pH 4. The mixture was heated to 40°C and stirred for 1 hour. The solution was clarified with a sequential combination of 600 µm sieving, centrifugation and 1 µm glass fibre vacuum filtration. The clarified extract was made up to 50% saturation ammonium sulphate and stirred gently over ice for 1 hour. The resultant precipitate was harvested by centrifugation, washed with a 55% saturated ammonium sulphate solution and re-harvested. The precipitate was resuspended in 60% aqueous ethanol (1 L) and left overnight at 4°C. The solution was warmed to 40°C with stirring for 1 hour and the supernatant containing the protein of interest obtained by centrifugation and 1 µm filtration. Bowman Birk inhibitor was precipitated by the addition of ethanol (500 ml) and harvested by centrifugation. The pellet containing the inhibitor was washed with ethanol, re-harvested and air dried. Crude BBI was further purified by immobilised nickel affinity and anion exchange chromatography before dialysis against pH 7.5 HEPES (10 mM), NaCl (100 mM) and sterile filtration. Purified BBI (900 mg) was judged to be >95% pure based on SDS-PAGE with Coomassie blue staining and densitometry. The inhibitor solution was freeze dried for inclusion in some OraCAb formulations.

**1.2** **Enzyme-Linked ImmunoSorbent Assay (ELISA) for detection of ovine IgG in hamster serum**

Coat ImmulonR 4HBX flat bottom microtiter plates (Thermo Scientific) were coated with serial 2-fold dilutions of hamster sera in Phosphate Buffered Saline (PBS). A 100 µl of each dilution was added per well. For positive control, 100μl of purified sheep IgG with concentration of 6.25mg/l was added to the well with highest IgG concentration. Two-fold dilutions were used for the rest of the wells for the positive control. The above dilutions for each sample were set in triplicates on the plates and incubate overnight at 4ºC. Plates were then washed three times with PBS containing 0.1% Tween 20 (PBST) and blocked with 2.5% foetal bovine serum in PBS (150µl/well) for 2 hours at 37ºC. Following the blocking, wells were washed thrice with PBST and 100µl of diluted (1/12,000) Donkey anti-Sheep IgG coupled to horse radish peroxidase (Sigma) were added to each well. After incubation for an hour at 37ºC, plates were washed three times with PBST. Development was carried out by adding 100 µl/well of substrate solution 3,3′,5,5′-tetramethylbenzidine (TMB; Abcam) followed by incubation of the plates in the dark for 10 minutes. Reaction was stopped by adding 50 µl /well of 1M HCl (Merck). Absorption was determined by reading each well at 450 nm and 690 nm in a POLARstar Omega reader (MBG Labtech).

# 2 Supplementary Figures

## 2.1 Supplementary Figure 1

##
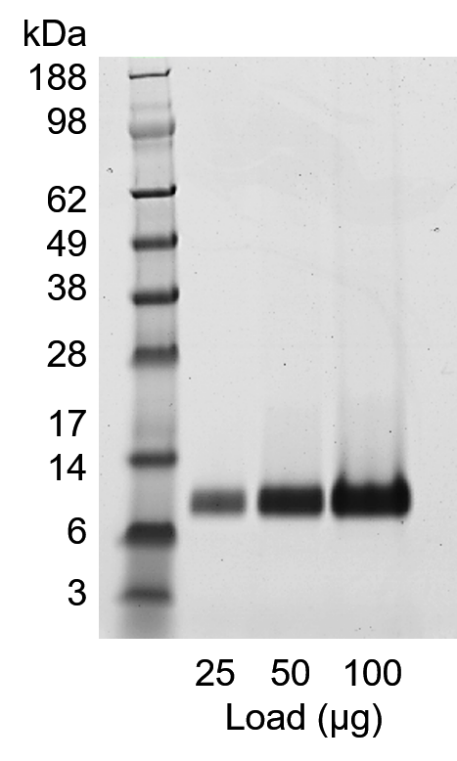


## Supplementary Figure 1. SDS-PAGE purified Bowman Birk Inhibitor.

## 2.2 Supplementary Figure 2

**Supplementary Figure 2. Lack of infiltration of ovine IgG in blood circulation of hamsters infected with *C. difficile* and treated with OraCAb.**

**
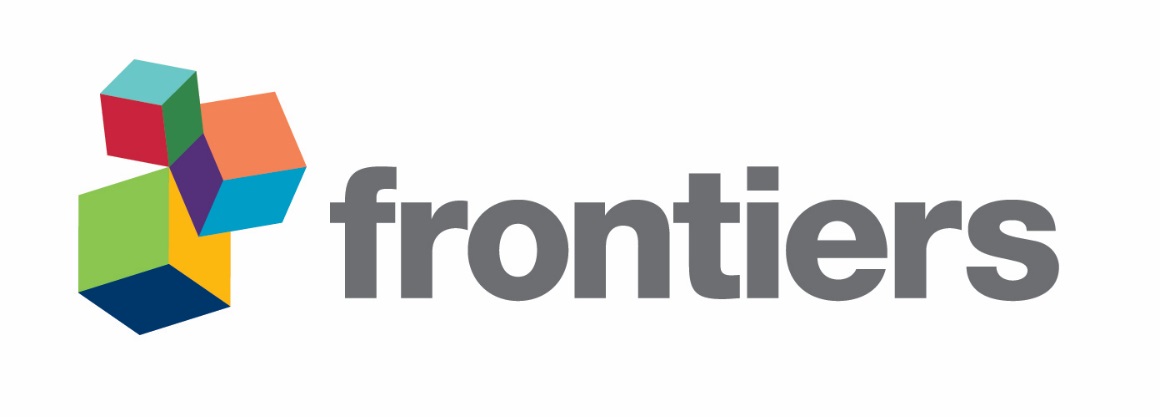
**
